# Case Report: Rare factor V inhibitor found in a patient with ulcerative colitis during latent tuberculosis infection

**DOI:** 10.3389/fmed.2026.1755053

**Published:** 2026-06-02

**Authors:** Yaodong Wang, Na Li, Xiaoxin Li, Jinchun He

**Affiliations:** 1 Laboratory Medicine Center, The Second Hospital of Lanzhou University, The Second School of Clinical Medicine of Lanzhou University, Lanzhou University, Lanzhou, Gansu, China; 2 Department of General Surgery, The Second Hospital of Lanzhou University, The Second School of Clinical Medicine of Lanzhou University, Lanzhou University, Lanzhou, Gansu, China; 3 Department of Laboratory Medicine, The First Hospital of Lanzhou University, The First School of Clinical Medicine of Lanzhou University, Lanzhou University, Lanzhou, Gansu, China

**Keywords:** coagulation factor V inhibitor, immunoregulation, latent tuberculosis infection, mixing studies, ulcerative colitis

## Abstract

Acquired factor V deficiency (AFVD) is a rare disease characterized by a relative deficiency of coagulation factor V (FV) due to the presence of internal FV inhibitors. Patients may exhibit a range of symptoms from asymptomatic to severe bleeding. The heterogeneity of AFVD among clinical conditions makes its diagnosis challenging. Infection is a common secondary cause of FV inhibitors. This article reported a case of a patient with ulcerative colitis (UC) in the latent tuberculosis infection (LTBI) stage, who had abnormal initial screening of coagulation function. Traditional treatment strategies targeting the primary disease and empirical anti-infection therapy did not improve the patient's condition. It was not until the laboratory reported high-titer FV inhibitors followed by the clinical team providing immunomodulatory treatment with intravenous immunoglobulin and preventive anti-tuberculosis therapy, which successfully led to the elimination of FV inhibitors and significant improvement in the bleeding and infection symptoms of the primary disease. This article reviews relevant literature to provide ideas for the clinical diagnosis and treatment of rare factor inhibitors. To our knowledge, this is the first report of FV inhibitor in patients with UC during LTBI, as well as the first observation of a benefit in using immunoglobulin regimen to clear cycle inhibitors.

## 1 Introduction

Latent tuberculosis infection (LTBI) is defined as a state in which the body maintains an immune response to Mycobacterium tuberculosis antigen stimulation without clinical manifestations of active tuberculosis. Over 90% of LTBI cases will not progress to clinical tuberculosis if treated early with preventive antituberculosis therapy. Although no gold standard currently exists for diagnosing LTBI, tuberculin skin tests and gamma interferon release assays remain widely accepted for its identification (1). It is well known that conventional drugs for treating Mycobacterium tuberculosis infection, such as rifampicin, isoniazid, and ethambutol, can cause drug-induced liver injury (2). This further interferes with the synthesis of numerous coagulation factors in the liver, leading to an increased risk of bleeding (3). Therefore, caution is warranted when using these anti-tuberculosis drugs in patients with tuberculosis who also have other bleeding disorders, such as ulcerative colitis (UC) and/or high-titer factor V (FV) inhibitor.

FV is a coagulation factor in the common pathway of the coagulation cascade. Acting as a cofactor for Factor X (FX), it forms a complex with calcium ions and phospholipids to activate downstream factors, participating in critical physiological hemostasis processes. If FV activity is reduced or inhibited, the risk of bleeding inevitably increases. FV inhibitors function similarly to anti-FV antibodies, suppressing FV activity despite adequate FV levels in the body. Under the circumstances, appropriate therapeutic approach involves eliminating inhibitors through immunosuppressive or immunomodulatory strategies, rather than supplementing with clotting factors or fresh frozen plasma (FFP) (4–6). In addition, the detectable rate of factor inhibitors such as FV inhibitor in laboratory is usually low. This is because they mainly cause false changes in screening items for hemostasis and coagulation function, including prolonged activated partial thromboplastin time (APTT) and prothrombin time (PT), false positive lupus anticoagulant (LA), and low levels of coagulation factor profiles. If only these false positive results are reported to the clinical department, it will mask the true cause, lead clinical diagnosis and treatment in a wrong direction, delay the diagnosis and treatment of the disease, and even lead to disastrous consequences. Therefore, for unexplained prolongation of APTT and/or PT, the presence of inhibitors can usually be preliminarily judged through the APTT/PT mixing studies (7). The Rosner index (when over 15%) has been used to predict the presence of an inhibitor or a LA (8).

UC is a recurrent inflammatory bowel disease primarily characterized by abdominal pain, recurrent diarrhea, and even mucus-containing bloody stools. In recent years, its incidence and prevalence have shown an upward trend. 5-aminosalicylic acid derivatives (e.g., mesalazine) remain the first-line treatment for mild to moderate disease. If these agents prove ineffective, escalation to immunosuppressants and biologics (e.g., infliximab) is warranted (9, 10). However, note that patients treated with infliximab have an increased risk of serious infections. Therefore, patients should be screened for LTBI before and during treatment with this drug. If the test result is positive, LTBI should be treated prior to initiating therapy (11).

The current study describes a case of UC with LTBI in which a rare FV inhibitor was detected. Treatment to clear the inhibitor showed significant improvement in the patient's primary symptoms, including anemia and bleeding. Combined with related literatures, we aim to provide insights for the clinical diagnosis and treatment of rare factor inhibitors.

## 2 Case description

A 48-year-old Han Chinese male farmer was admitted for further management with an initial diagnosis of UC, who had received no prior treatment before admission to our hospital. In terms of clinical manifestations, he presented with intermittent abdominal pain and diarrhea (7–8 times/day) accompanied by mucopurulent bloody stools for over 1 month. Physical examination revealed hyperactive bowel sounds at 7 times per min. No petechiae or ecchymoses were observed on skin or mucous membranes. The patient reported no family history of bleeding disorders, no rheumatic diseases, no recent use of anticoagulants such as warfarin, no exposure to rodenticides, and no history of blood transfusions. Abdominal ultrasound revealed thickened colonic and rectal walls with luminal narrowing throughout the colon. Transrectal colonoscopy with tissue biopsy demonstrated multiple colorectal ulcers (consider infection). Key laboratory results are summarized in Table 1. Hematologic laboratory findings indicated moderate normocytic normochromic anemia. And fecal occult blood test was positive, APTT and PT were markedly prolonged, while thrombin time (TT) was normal. To identify the cause of prolongation and assess surgical bleeding risk, APTT and PT mixing studies were initiated, both of which failed to correct the abnormalities. LA testing yielded positive results, suggesting potential high-titer LA presence, though the possibility of factor inhibitor could not be excluded. Coagulation factor activity tests showed a general decrease. Ultimately, factor inhibitor analysis confirmed the presence of high-titer FV inhibitor. Laboratory reports also indicated that such high-titer FV inhibitor simultaneously caused false-positive LA test results and interfered with other coagulation factor activity assays, leading to falsely low results.

**Table 1:** Key laboratory screening results.

Test	Patient value	Reference interval
HGB, g/L	89	130–175
PLT, 10^9^/L	491	125–350
ALB, g/L	25.1	40-55
PT, s	34.7	9.4–12.5
APTT, s	79.5	25.4–38.4
Fibrinogen, g/L	4.69	2.0–5.0
TT, s	14.4	10.3–17.6
D-dimer, μg/mL	0.28	< 0.5

HGB, hemoglobin; PLT, platelets; ALB, albumin; PT, prothrombin time; APTT, activated partial thromboplastin time; TT, thrombin time.

## 3 Clinical course

During hospitalization, clinical management included administration of a combination of live lactobacilli and Bacillus subtilis-Enterococcus faecalis to regulate intestinal flora. Nutritional support was provided through infusion of vitamin B6, vitamin C, glucose, albumin (ALB), and enteral nutrition. Water-electrolyte and acid-base balance were adjusted, and mesalazine was administered to treat the primary disease. On the first day of admission (January 13), initial screening results including coagulation function were abnormal (APTT 79.5 s, PT 34.7 s, hemoglobin 89 g/L, ALB 25.1 g/L). Accordingly, the clinician gave vitamin K intramuscularly (IM) daily (QD). Follow-up coagulation tests on a second day (January 15) showed no improvement in APTT and PT (APTT 73.2 s, PT 32.1 s). Viral-inactivated FFP was therefore requested and transfused at 200 mL on both the 5th and 6th days of hospitalization (January 17 and 18) to replenish coagulation factors. Concurrently, Vitamin K 110 mg QD IM was maintained. Subsequent re-evaluation showed APTT 77.8 s, PT 34.6 s, hemoglobin 81 g/L, ALB 25 g/L, highlighting a disappointing improvement.

Such patient experienced a transient fever on the night of the third hospitalization day with temperature reaching 38.6°C. Infection-related markers showed significantly elevated C-reactive protein (73 mg/L) and mildly elevated procalcitonin (0.25 ng/mL), thus empirical antimicrobial therapy with ceftriaxone was provided. Screening for tumor markers and pathogen testing—including influenza virus, SARS-CoV-2, TORCH panel, EBV, HIV, syphilis, HBV, HCV, and Clostridium difficile—all returned negative results. However, a positive result was reported in the tuberculosis-specific gamma interferon release assay. Since no abnormalities were shown in chest imaging examination and as a result, the condition was considered LTBI.

**Figure 1:**
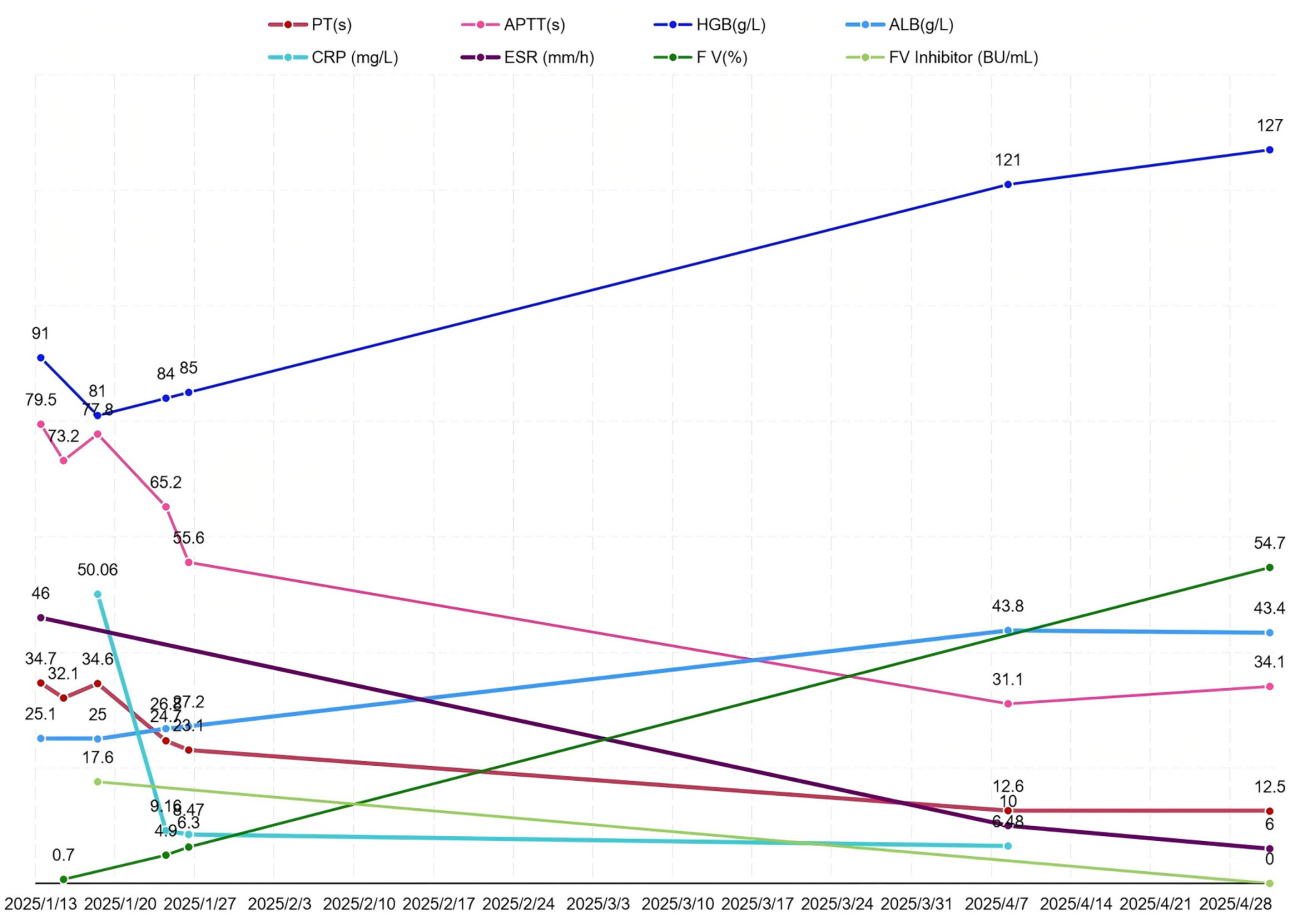
Trend of key indicators before and after administration of FV inhibitor.

Six days after the initial abnormal coagulation screening (January 19), laboratory finally reported high-titer FV inhibitor. Clinicians immediately discontinued vitamin K, FFP, and ALB transfusions. Concurrently, intravenous immunoglobulin (IVIG) therapy at 10 g QD was started for inhibitor clearance. Five days later, follow-up coagulation tests on January 24 saw declining APTT (65.2 s) and PT (24.7 s). Continue for 2 days, his condition and coagulation parameters improved further (APTT 55.6 s, PT 23.1 s). Similarly, infection-related markers also largely normalized (C-reactive protein 8.5 mg/L, procalcitonin 0.11 ng/mL) as well as the general condition improved. Finally, he was discharged on the 14th day of hospitalization (January 26). At the 2- and 3-month follow-up visits respectively after discharge, his coagulation screening indicators, infection markers, anemia, and hypoalbuminemia had all fully recovered to normal. Of course, the final test for FV inhibitor was also conducted in the 3rd month after discharge, with results showing no detectable levels. Trend of key indicators before and after FV inhibitor administration was shown in Figure 1.

## 4 Laboratory analysis approach for FV inhibitor

### 4.1 Initial screening of coagulation function

The patient's initial screening of coagulation routine tests showed that both APTT and PT were prolonged simultaneously, but TT and plasma fibrinogen were normal. This is commonly seen in cases of vitamin K-dependent coagulation factor deficiency, taking warfarin or rat poison poisoning, as well as liver and kidney dysfunction. However, it is extremely rare in cases of coagulation factor deficiency in the common pathway (such as FV), or factor inhibitors in the common pathway (such as FV inhibitor).

### 4.2 APTT and PT mixing studies

As shown in Table 2, during immediate studies, the Rosner indexes for APTT and PT in 1:1 mixing were 24.7 and 42.2%, respectively. Correspondingly, after incubation at 37°C for 2 h, their Rosner indexes were 53.9 and 59.8%, respectively. This indicates that neither mixing was corrected. At the same time, a significant prolongation was also observed in the above-mentioned mixed results after incubation (for both APTT and PT) compared to that the immediate results. This indicates the presence of time- and temperature-dependent inhibitors. Therefore, the following three possible situations were considered: (1) Although coagulation factor VIII (FVIII) inhibitors are the most common inhibitors with time-temperature-dependent characteristics, they were excluded in this case because they did not match the patient's prolonged PT; (2) Inhibitors of common pathway factors, such as FX inhibitors, FV inhibitors, and coagulation factor II (FII) inhibitors; (3) Considering that a small portion of lupus anticoagulants have time-temperature-dependent characteristics and that LA positivity is relatively common in clinical practice, it could not be excluded for the time being.

**Table 2:** APTT and PT mixing studies.

APTT mixing studies		PT mixing studies	
Items	Results	Items	Results
APTT1	73.2 s	PT1	32.0 s
APTT2	31.7 s	PT2	10.5 s
APTT3	49.8 s	PT3	24.0 s
RI (In immediate)	24.73%	RI (In immediate)	42.19%
APTT4	74.9 s	PT4	33.1 s
APTT5	33.8 s	PT5	11.0 s
APTT6	74.2 s	PT6	30.8 s
RI (37°C for 2h)	53.9 %	RI (37°C for 2h)	59.8 %
[(APTT6–APTT3)/APTT3]^*^100%	49.0%	[(PT6–PT3)/PT3]^*^100%	28.3%

APTT, activated partial thromboplastin time; PT, prothrombin time; RI, rosner Index; APTT1 or PT1, The patient's APTT or PT results at the immediate time; APTT2 or PT2, APTT or PT results in normal pooled plasma (NPP) at the immediate time; APTT3 or PT3, APTT or PT results in 1:1 mixing with NPP at the immediate time; APTT4 or PT4, APTT or PT results after incubating the patient's plasma at 37°C for 2 h; APTT5 or PT5, APTT or PT results after incubating NPP at 37°C for 2 h; APTT6 or PT6, APTT or PT results after mixing patient plasma with NPP at a 1:1 ratio and incubating at 37°C for 2 h.

### 4.3 LA detection

Applying the diluted viper venom test, LA was detected in the patient's plasma (results shown in Table 3). Both the screening and confirmation tests failed, entering the mixed test procedure. This involved mixing with normal pooled plasma at a 1:1 ratio, followed by screening and confirmation tests, yielding results of 152.5 s and 91.2 s, respectively. The standardized ratio result was positive at 1.49. Current interpretation suggests either extremely high-titer LA or a common pathway inhibitor, though further testing is required for definitive identification.

**Table 3:** LA test results.

Items	Patient's plasma	Patient's plasma mixed 1:1 with NPP
dRVVT-S (s)	failed	152.5
S-ratio	/	5.05
dRVVT-C (s)	failed	91.2
C-Ratio	/	3.38
TR	/	1.49

The dRVVT-S and dRVVT-C values for NPP from our laboratory were 30.2 s and 27.0 s, respectively. LA, lupus anticoagulant; NPP, normal pooled plasma; dRVVT, dilute Russell's viper venom time; dRVVT-S, screening test for dRVVT; dRVVT-C, confirming test for dRVVT; S-Ratio, The ratio of the patient's dRVVT-S to the NPP's dRVVT-S; C-Ratio, The ratio of the patient's dRVVT-C to the NPP's dRVVT-C; TR, The ratio of S-Ratio to C-Ratio.

### 4.4 Coagulation factors activity assay

**Figure 2:**
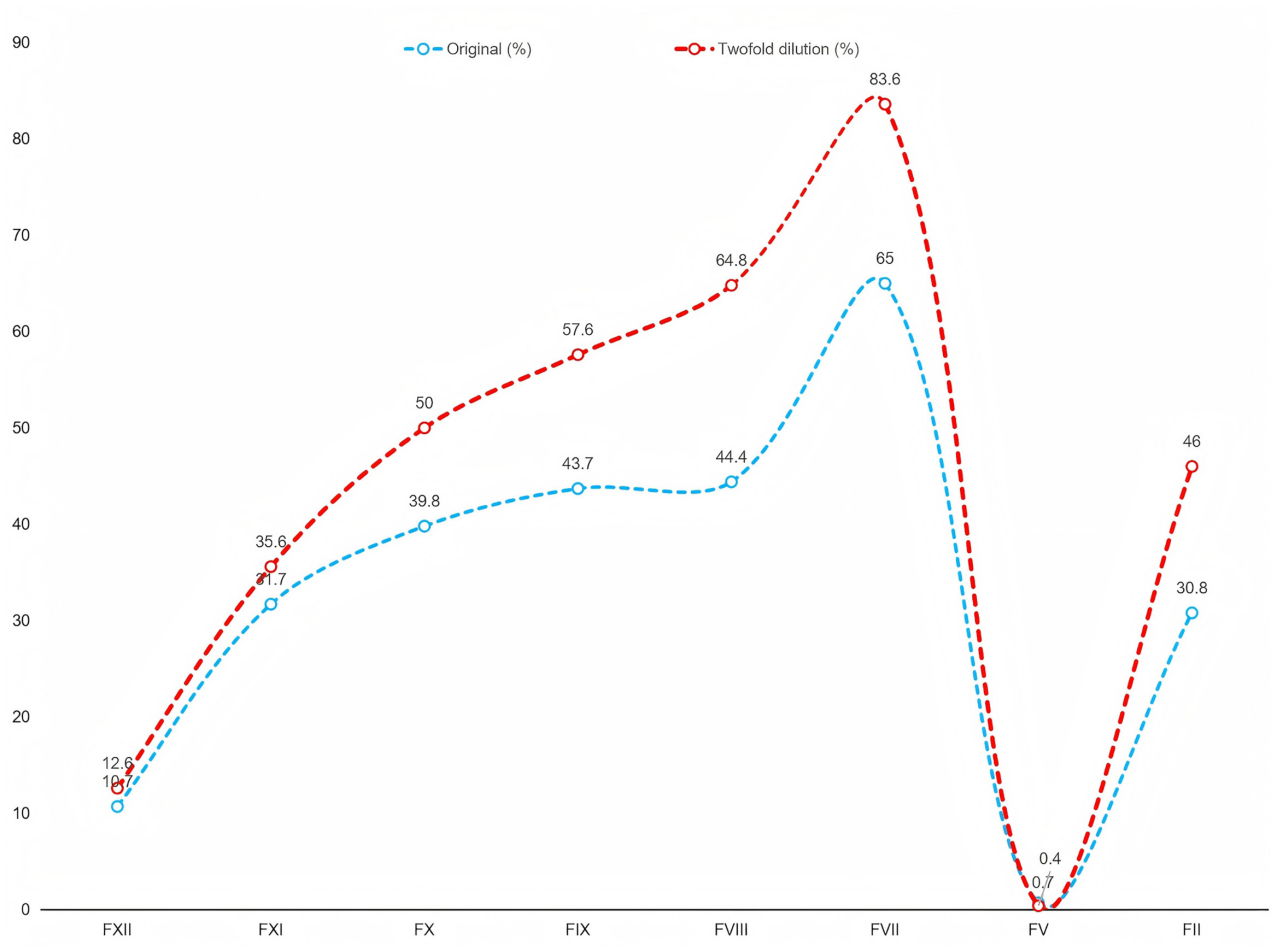
Changes in the coagulation factor profile before and after dilution.

Coagulation factors activity testing of the patient plasma revealed that coagulation factors XII (FXII), XI (FXI), X, IX (FIX), VIII, VII, V, and II were 10.7, 31.7, 39.8, 43.7, 44.4, 65, 0.7, and 30.8%, respectively. Activity levels were generally low with FV being the lowest which consistent with the characteristic widespread interference of inhibitors or LA in coagulation factor assays. Serial twofold dilutions are considered crucial for distinguishing factor inhibitors from LA. Accordingly, subsequent factors activity assay after twofold dilutions revealed that FV activity remained extremely low at 0.4%, while all other factors showed markedly elevated activity. Changes in the coagulation factor profile before and after dilution were shown in Figure 2. This strongly suggests the presence of a FV inhibitor.

### 4.5 Factor inhibitor analysis

Applying the Bethesda assay for FV inhibitor titer analysis with the result of 17.6 BU/mL indicates the presence of high-titer FV inhibitor (Table 4). Simultaneously, no FXII inhibitor was detected, indirectly supporting the absence of LA. Indeed, during hospitalization, such patient underwent antiphospholipid antibody panel testing (including anticardiolipin antibodies and anti-β2-GP1 antibodies), which yielded negative results. Combined with the negative FXII inhibitor test, this conclusively indicated a false-positive LA result in this patient, attributable to interference from the FV inhibitor.

**Table 4:** Analysis of coagulation factor inhibitors.

Factors	Control (%)	Logarithmic dilutions					Inhibitors titer (BU/mL)
		**1:2 (%)**	**1:4 (%)**	**1:8 (%)**	**1:16 (%)**	**1:32 (%)**	
FV	38.9	5.5	8.1	11.8	17.9	26.6	17.6
FXII	27.1	28.4	28.4	–	–	–	0

FV, coagulation factors V; FXII, coagulation factor XII.

## 5 Discussion

Acquired FV inhibitors are essentially IgG-class immunoglobulins targeting the phosphatidylserine binding site of FV (12, 13). According to previous literature reports, the production of factor inhibitors is often associated with factors such as infection, drugs, blood product transfusion, malignant tumors, and autoimmune diseases (14). Considering such patient's clinical changes before and after IVIG treatment, along with the consistent trend between circulating inhibitor levels and improvements in infection symptoms and markers post-IVIG administration, it is reasonable to infer that the production of inhibitors in this patient is associated with LTBI. Inflammatory bowel disease, specifically UC, is a multifactorial disorder involving interactions between infection and immunity, essentially representing an abnormal immune response directed against the host's own gut microbiota (15). Therefore, we hypothesize that IVIG treatment, while targeting FV inhibitors, may also confer therapeutic benefits for the underlying UC. Indeed, during the second follow-up period, as the patient showed no signs of infection, infliximab was initiated to modulate the immune response in UC.

To elucidate the mechanism underlying the development of FV inhibitors in UC patients against the background of LTBI, it is necessary to construct a multi-factorial pathogenic model. In essence, this phenomenon does not stem from a single mechanistic pathway. Rather, it arises from an autoimmune dysregulation triggered by the synergistic effects of “molecular mimicry” and “bystander activation” under the combined influence of chronic inflammation, latent infection, and potential immune interventions (16–20). In fact, under normal physiological conditions, the immune system is tolerant to its own antigens (such as FV). However, patients with UC combined with LTBI are already in a state where their immune tolerance has been weakened. UC is a chronic inflammatory disease of the intestine and is inherently related to abnormal immune regulation. When the intestinal barrier is damaged, epitopes will spread, that is, the originally isolated self-antigens are exposed to the immune system, laying the groundwork for subsequent autoimmune reactions (21, 22). And the tuberculosis bacteria are in a dormant but metabolically active state within the granuloma, and their antigens (such as ESAT-6, CFP-10, Ag85, etc.) are continuously and at a low level released, maintaining the memory of specific T- and B-cells (23).

On one hand, molecular mimicry plays a pivotal triggering role, representing the most direct pathogenic hypothesis. This postulates that certain antigenic epitopes of Mycobacterium tuberculosis exhibit localized structural similarity with the protein structure of FV. When LTBI is reactivated by certain factors, or merely due to increased antigen load, antibodies or T-cell receptors targeting mycobacterial antigens—such as members of the heat shock protein family like Ag85 or Hsp65—may inadvertently recognize and attack FV owing to such structural resemblance (24). Moreover, recent bioinformatic analyses have further corroborated that certain mycobacterial proteins indeed share homologous sequences with human coagulation-related proteins. Particularly in the context of UC, characterized by a highly activated immune state, such low-affinity cross-reactivity is more readily amplified (17).

On the other hand, bystander activation serves as a key synergistic mechanism. Even in the absence of direct molecular mimicry, a robust inflammatory milieu alone can trigger autoimmunity. During active UC, massive amounts of pro-inflammatory cytokines such as TNF-α, IL-6, and IFN-γ are released (25). Granulomas localized near latent infectious foci may undergo subtle disruption under the influence of UC-associated systemic inflammation, leading to substantial release of tuberculosis antigens (25). This intense inflammatory environment, particularly elevated TNF-α levels, contributes to nonspecific activation of T-cell and B-cell clones (26). Among these, previously quiescent autoreactive B cells targeting FV may become activated, resulting in the production of anti-FV antibodies. Moreover, while the initial immune response may be directed against mycobacterial antigens, inflammation-induced tissue damage—such as vascular endothelial injury—can expose sequestered FV antigens. This leads to subsequent recognition and targeting of these newly exposed self-antigens by the immune system (25).

Certainly, in addition to the above theoretical analysis of the possible associations between FV inhibitors, UC, and LTBI, we also reviewed and compared similar cases reported in the literature, as shown in Table 5.

**Table 5:** Summary of similar case reports on coagulation abnormalities in diseases near LTBI/UC.

Source	Patient profile	Underlying diseases near LTBI/UC	Type of coagulation abnormality near factor V	Clinical outcome/notes
Taillan et al. (42)	2 patients	1 with celiac disease, 1 with Crohn's disease	Factor V inhibitor	Inhibitors appeared during the active phase of intestinal disease and disappeared during remission; no bleeding events occurred.
Kyriakou et al. (43)	15 out of 511 patients with gastrointestinal diseases developed coagulation factor inhibitors	302 UC, 112 Crohn's disease, 82 gastrointestinal cancer, and 15 lymphoma	1 case of Factor V inhibitor (from the UC group); others were inhibitors against Factors VIII, IX, X, XI, and XII	Only 1 patient with a Factor VIII inhibitor experienced severe bleeding; all cases resolved following immunosuppressive therapy or tumor resection.
Skowroński et al. (44)	31-year-old male	Pulmonary tuberculosis	Factor V Leiden mutation	Pulmonary embolism and pulmonary infarction; condition improved after anti-tuberculosis treatment and anticoagulation therapy.
Ogilvie et al. (45)	23-year-old female	UC	Factor V Leiden mutation and prothrombin gene mutation	Developed right ventricular thrombus and pulmonary embolism following the use of infliximab (anti-TNF-α)
Lerolle et al. (46)	65-year-old male	Pulmonary tuberculosis	Factor V inhibitor	Inhibitor was discovered 12 years after it had received isoniazid, streptomycin and PAS treatment for tuberculosis.
Aliaga et al. (47)	29-year-old male	Pulmonary tuberculosis	Factor V inhibitor	Bleeding tendency may be caused by antibodies against clotting factors, and the condition can improve after anti-tuberculosis chemotherapy.
Takase et al. (48)	Middle-aged male	Pulmonary tuberculosis	Factor V inhibitor	Not mentioned/Not screened

LTBI, latent tuberculosis infection; UC, ulcerative colitis.

In reality, clinical laboratories play a pivotal role, as they must proactively investigate potential interfering factors and initiate corrective testing for initial screening results showing APTT prolongation with PT prolongation. This involves conducting further analyses to ultimately determine the cause and provide a risk assessment (27, 28).

Among the numerous causes leading to APTT/PT prolongation, common pathway coagulation factor deficiencies are most prevalent. These primarily involve deficiencies in factors II, VII, IX, and X due to vitamin K deficiency, carrying a high bleeding risk. Clinically, vitamin K supplementation is often administered to promote coagulation factor production (7). Therefore, following the initial abnormal screening results in this case report's coagulation panel, clinicians empirically administered with vitamin K to indirectly supplement coagulation factors until laboratory reports confirmed a positive FV inhibitor. The 2023 ACR/EULAR scoring system for antiphospholipid syndrome (APS) diagnosis incorporates a single positive LA test result, assigning 5 points for persistent LA positivity (29). It is well-known that APS carries a high risk of thrombosis, with catastrophic APS occasionally reported (30, 31). Consequently, LA has become an increasingly important screening marker for APS in clinical practice. A positive LA test is generally observed with isolated APTT prolongation, unless extremely high LA titers cause simultaneous prolongation of both APTT and PT (32). Literature reports indicate that some LA also exhibits time-temperature-dependent characteristics (33, 34). Thus, in this case, LA testing appears to be an insurmountable confounding step in identifying the FV inhibitor pathway. If laboratories skip the correction test step and directly perform LA testing reporting a positive result, the true situation (coagulation factor deficiency or inhibitor presence) would be obscured, potentially misleading clinicians into administering incorrect treatment. Parallel factor dilution assays can distinguish between LA and factor inhibitors by diluting the sample to attenuate interference with factor assays. In the presence of LA, factor levels generally normalize with increasing dilution titers. Conversely, with high-titer inhibitors, the corresponding factor level remains low while others normalize.

Clinical manifestations of FV inhibitor exhibit high heterogeneity, potentially presenting with severe bleeding or no bleeding symptoms, manifesting only as coagulation parameter abnormalities (35). This may be related to the different binding sites of inhibitors on FV (14). In fact, more bleeding events have been reported as in this case. The current patient experienced intermittent rectal mucosal bleeding for over a month, presenting with mild to moderate anemia at admission. We observed that clinical administration of FFP did not improve plasma protein levels. Only after laboratory confirmation of a positive FV inhibitor test and initiation of IVIG for immunomodulatory therapy did the patient's plasma protein levels normalize and anemia resolve as the inhibitor waned. Therefore, it is hypothesized that the patient's FV inhibitor competitively binds to the phosphatidylserine binding site (i.e., the C2 domain) of FV (12, 14, 36), inhibiting the formation of the activated FV-FX-Ca^2+^-phospholipid complex. This, in turn, suppresses the activation of downstream coagulation factors and the formation of fibrin monomers, ultimately leading to bleeding events. However, some FV inhibitor-positive patients exhibit no bleeding. This may relate to FV stored in platelet α-granules: upon platelet activation, FV released at the bleeding site can participate in the amplification of the coagulation cascade before being interfered with by the inhibitor, thereby effectively promoting hemostasis (14, 37).

Treatment for patients with positive FV inhibitors requires comprehensive consideration based on the presence and severity of bleeding, along with the underlying disease. Asymptomatic patients generally do not require treatment, but elimination of the inhibitor through immunosuppressive agents may be beneficial. For example, De Raucourt et al. reported that in a patient without bleeding symptoms, FV inhibitors resolved within 1 week following IVIG immunosuppressive therapy (4). In contrast, Gadelha et al. reported that in a patient with FV inhibitor but no bleeding symptoms, no intervention was administered. After 29 months of follow-up, the inhibitor persisted (38). For patients with clinical bleeding and FV inhibitors, treatment with blood products like prothrombin complex concentrates or recombinant human factor VIIa (rFVIIa) may be challenging due to the inhibitor's direct interference with the common pathway of coagulation factor activation by binding to the FX-FV-Ca^2+^-phospholipid complex (14). Furthermore, platelet transfusion therapy for bleeding caused by FV inhibitors has been reported to achieve efficacy rates ranging from 35 to 71% (5, 39). However, most literature reports on FV inhibitor treatment involve combination strategies (5), not the sole use of a single immunosuppressive agent or blood product, despite this case patient receiving only IVIG. For instance, George et al. reported a patient with gastric bleeding who achieved resolution of inhibitors within 3 weeks through combined use of FFP, vitamin K, platelets, IVIG, glucocorticoids, and cyclophosphamide (40). Another case involving a patient with ecchymoses and abdominal hematoma was treated with rFVIIa, IVIG, rituximab, and glucocorticoids for 4 weeks, resulting in clearance of circulating inhibitors (41). Why, then, did this patient not receive glucocorticoids or rituximab to treat circulating inhibitors after laboratory confirmation of high-titer FVII inhibitors? The primary reasons we considered were: (1) the patient's elevated blood glucose levels, where corticosteroid administration could exacerbate glycemic burden, and (2) the presence of tuberculosis infection, where immunosuppressive agents like rituximab and corticosteroids might further compromise an already compromised host defense system.

A limitation of this report is that we assessed the improvement in the patient's inhibitors solely by measuring his APTT and PT, without continuously monitoring the trend in inhibitor titer levels. We only determined his FV inhibitor titer during his third follow-up visit (3 months later), leaving us without data to evaluate the earliest possible time of inhibitor clearance.

In summary, FV inhibitors are extremely rare and can easily mislead clinicians due to their potential to cause falsely decreased levels in coagulation factor assays and false-positive results in LA testing. Therefore, laboratories must strictly adhere to correction protocols to accurately identify FV inhibitors.

## Data Availability

The original contributions presented in the study are included in the article/supplementary material, further inquiries can be directed to the corresponding author.
